# Baseline Preferences for Daily, Event-Driven, or Periodic HIV Pre-Exposure Prophylaxis among Gay and Bisexual Men in the *PRELUDE* Demonstration Project

**DOI:** 10.3389/fpubh.2017.00341

**Published:** 2017-12-15

**Authors:** Stefanie J. Vaccher, Christopher Gianacas, David J. Templeton, Isobel M. Poynten, Bridget G. Haire, Catriona Ooi, Rosalind Foster, Anna McNulty, Andrew E. Grulich, Iryna B. Zablotska, On Behalf of the PRELUDE Study Team, Andrew Carr

**Affiliations:** ^1^The Kirby Institute, UNSW Sydney, Sydney, NSW, Australia; ^2^RPA Sexual Health, Sydney Local Health District and Central Clinical School, The University of Sydney, Sydney, NSW, Australia; ^3^Western Sydney Sexual Health Centre, Western Sydney Local Health District, Parramatta, NSW, Australia; ^4^Centre for Infectious Diseases and Microbiology, Westmead Clinical School, University of Sydney, Sydney, NSW, Australia; ^5^Clinic 16 Northern Sydney Sexual Health, Royal North Shore Community Health Centre, St Leonards, NSW, Australia; ^6^Sydney Sexual Health Centre, Sydney, NSW, Australia; ^7^School of Public Health and Community Medicine, UNSW Sydney, Sydney, NSW, Australia

**Keywords:** pre-exposure prophylaxis, non-daily dosing, event-driven pre-exposure prophylaxis, periodic pre-exposure prophylaxis, HIV, gay and bisexual men, dosing preferences

## Abstract

**Introduction:**

The effectiveness of daily pre-exposure prophylaxis (PrEP) is well established. However, there has been increasing interest in non-daily dosing schedules among gay and bisexual men (GBM). This paper explores preferences for PrEP dosing schedules among GBM at baseline in the *PRELUDE* demonstration project.

**Materials and methods:**

Individuals at high-risk of HIV were enrolled in a free PrEP demonstration project in New South Wales, Australia, between November 2014 and April 2016. At baseline, they completed an online survey containing detailed behavioural, demographic, and attitudinal questions, including their ideal way to take PrEP: daily (one pill taken every day), event-driven (pills taken only around specific risk events), or periodic (daily dosing during periods of increased risk).

**Results:**

Overall, 315 GBM (98% of study sample) provided a preferred PrEP dosing schedule at baseline. One-third of GBM expressed a preference for non-daily PrEP dosing: 20% for event-driven PrEP, and 14% for periodic PrEP. Individuals with a trade/vocational qualification were more likely to prefer periodic to daily PrEP [adjusted odds ratio (aOR) = 4.58, 95% confidence intervals (95% CI): (1.68, 12.49)], compared to individuals whose highest level of education was high school. Having an HIV-positive main regular partner was associated with strong preference for daily, compared to event-driven PrEP [aOR = 0.20, 95% CI: (0.04, 0.87)]. Participants who rated themselves better at taking medications were more likely to prefer daily over periodic PrEP [aOR = 0.39, 95% CI: (0.20, 0.76)].

**Discussion:**

Individuals’ preferences for PrEP schedules are associated with demographic and behavioural factors that may impact on their ability to access health services and information about PrEP and patterns of HIV risk. At the time of data collection, there were limited data available about the efficacy of non-daily PrEP schedules, and clinicians only recommended daily PrEP to study participants. Further research investigating how behaviours and PrEP preferences change correspondingly over time is needed.

**Trial registration:**

ClinicalTrials.gov NCT02206555. Registered 28 July 2014.

## Introduction

The effectiveness of once-daily oral tenofovir disoproxil fumarate and emtricitabine as pre-exposure prophylaxis (PrEP) for the prevention of HIV has been clearly demonstrated (1). Uptake of PrEP has been constrained by its high cost which, together with concerns about side effects and the perceived burden of taking a pill every day, has delayed licencing in many settings (2–5). However, since 2014, PrEP use has increased substantially, particularly in developed settings with large populations of gay and bisexual men (GBM) populations such as the United States (6) and Australia (7). As the population of PrEP users diversifies, more HIV prevention choices are required to ensure people with varying needs, concerns, and risk patterns can access the most suitable strategy for their individual situation. This has led to calls for new non-daily dosing regimens of oral PrEP to be explored (8–10).

Non-daily PrEP schedules can be conceptually divided into two different categories: *event-driven PrEP*, where pills are taken “on demand” around the time of risk events; and *periodic PrEP*, where pills are taken daily during periods or “seasons” of risk. For instance, event-driven PrEP may suit someone with an HIV-positive partner where sex events could be planned in advance. Individuals who predominantly have risk events in specific defined periods, such as when travelling or at the time of large gay community events, may be better suited to periodic PrEP use. These non-daily regimens can be used to reduce costs and decrease the risk of short- or long-term side effects (11–14), but may be more complicated in terms of adherence (15).

Evidence has emerged to support an event-driven PrEP schedule in GBM, with the IPERGAY study reporting an 86% HIV risk reduction among participants taking a four-pill regimen around the time of sex events. This study confirmed that GBM at high risk of HIV can adhere to more complex PrEP dosing regimens and that these non-daily regimens are highly effective. The acceptability of alternative PrEP regimens has also been explored among heterosexual women (16, 17). However, HIV infections have been recorded in women taking fewer than six PrEP pills per week, due to lower drug concentrations in vaginal—compared to rectal—tissues (18, 19). In GBM, taking only four pills per week is associated with a 96% reduction in HIV incidence (20). As such, current recommendations for non-daily PrEP regimens are specifically targeted towards men (21, 22).

In Australia, only daily PrEP is recommended in national guidelines (23). *PRELUDE* was the first demonstration study to provide free access to PrEP in New South Wales (NSW), Australia’s most populous state. It collected demographic, behavioural, and attitudinal data, including preferences for daily, event-driven, and periodic PrEP use, providing a more nuanced understanding of individuals’ PrEP needs. This paper aims to explore preferences for PrEP dosing and associated factors among GBM, as well as attitudes towards PrEP use in a cohort of early PrEP adopters in NSW, Australia.

## Materials and Methods

### Study Design and Participants

*PRELUDE* was an open-label demonstration project aimed at assessing the feasibility and acceptability of PrEP delivery among people at high risk of HIV in NSW, Australia. Overall, 98% of participants were GBM. Women with an HIV-positive partner looking to conceive naturally were eligible for enrolment in *PRELUDE*, but were not included in this analysis due to the different indication for PrEP.

The study protocol has been reported previously (24). Briefly, the baseline study visit included an assessment of HIV risk, detailed medical history, testing for HIV, sexually transmissible infections (STIs), and pregnancy (where applicable), and prescription of PrEP. Immediately after their visit, all participants were emailed a detailed demographic, behavioural, and attitudinal survey to be completed in private. If a response was not received within 3 days, two email reminders were sent, 1 week apart. All participants provided written informed consent before undertaking any study procedures, in accordance with the Declaration of Helsinki. The study was approved by St. Vincent’s Hospital Sydney Human Research Ethics Committee and registered under ClinicalTrials.gov (identifying number NCT02206555). No monetary incentives for participating in the study were provided.

### Data Collection

All data analysed in this paper are from the baseline behavioural survey, conducted online using the SurveyGizmo (Boulder, CO, USA) platform. This survey collected detailed sociodemographic information, as well as attitudinal and behavioural data. Participants’ date of birth was recorded in the clinical data collection system, and age at enrolment was calculated.

At baseline, participants were asked to select their ideal way to take PrEP out of the following three options: daily (one pill taken every day), event-driven (pills taken only around specific risk events), or periodic (daily dosing during periods of increased risk).

Questions about sexual partners and sex practices in the previous 3 months asked specifically about partner type (main regular, other regular, or casual), HIV status of partner, and the number and type of anal sex events (condom use, ejaculation, and being the insertive or receptive partner) with each partner. Using this information, a variable was created specifying the number of times a participant had engaged in receptive condomless anal intercourse (CLAI) with a regular or casual partner of HIV-positive or unknown status in the last 3 months. HIV transmission risk was assessed by asking participants about the partner type they were most concerned about acquiring HIV from, with multiple selections permitted.

Other behavioural practices, including drug use, were also examined. Participants were asked about their frequency of crystal methamphetamine use, and episodes of binge-drinking, which was defined as having consumed five or more alcoholic drinks in a row. Responses to each question collapsed into three categories (never, once a month, and more than once a month). Participants were also asked about having had group sex (never, once or twice, and three times or more) in the previous 3 months.

Three scales were included in the baseline survey to assess beliefs and attitudes: an HIV transmission risk scale, an HIV anti-retroviral (ARV) scale, and a scale where participants rated their perceived ability to adhere to medication (medication self-efficacy scale). Questions included in each of the three scales are shown in Table S1 in Supplementary Material. For each set of questions, a four-point Likert scale was used, with a score calculated for each individual if they answered at least one question within that questionnaire (“Strongly disagree” = 1, “Disagree” = 2, “Agree” = 3, and “Strongly Agree” = 4).

Finally, participants were asked if they had first heard about PrEP more than or less than 12 months before the study, or only at the time they were invited to enrol into the study.

### Statistical Analysis

The main outcome of interest in this study was participants’ baseline preferred PrEP dosing schedule. Multinomial logistic regression models were used to test for associations between preferred PrEP dosing schedule and other baseline variables, using a purposeful selection approach (25). Initial univariate associations were calculated using likelihood ratio tests, and variables were included in the multivariable model if *p* ≤ 0.25. Variables were removed one at a time, starting with the highest *p*-value, and were retained in the final multivariable model if they were associated with the outcome at a significance level of *p* ≤ 0.05. Goodness of fit was assessed using a normalised Pearson chi-square test (25). Odds ratios (OR) for univariate analysis and adjusted odds ratios (aOR) for multivariable analysis are presented with 95% confidence intervals (95% CI). Analyses were conducted using R (R Core Team, 2016), with multinomial logistic regression models built using the mlogit package (Croissant, 2013).

## Results

Between November 2014 and April 2016, 321 GBM and three transgender (two female-to-male and one male-to-female) individuals were enrolled in *PRELUDE*. Participants had a mean age of 37 years, were predominately of Anglo-Saxon descent (66%), and the majority (63%) had a university-level education. Of the 316 GBM (98%) who completed the baseline online behavioural survey, 315 provided a preferred PrEP dosing schedule and were included in this analysis. The majority (*n* = 207; 66%) of participants expressed a preference for daily dosing; 20% (*n* = 64) preferred periodic dosing and 14% (*n* = 44) preferred event-driven PrEP. Cohort characteristics, overall and by PrEP dosing schedule preference, are presented in Table 1.

**Table 1:** Baseline characteristics of gay and bisexual male participants with a preferred pre-exposure prophylaxis (PrEP) schedule at baseline.

Characteristic	PrEP preference			
	Total cohort (*N* = 315)^a^^,^^b^	Daily (*N* = 207)	Event-driven (*N* = 44)	Periodic (*N* = 64)
Age [mean (SD)]	36.9 (9.1)	36.4 (9.1)	37.7 (8.6)	38.2 (9.6)
Transmission risk questionnaire score [mean (SD)]^c^	2.9 (0.5)	2.8 (0.5)	2.9 (0.5)	2.9 (0.5)
HIV anti-retroviral (ARV) questionnaire score [mean (SD)]^c^	3.1 (0.4)	3.1 (0.4)	3.1 (0.3)	3.1 (0.3)
Self-efficacy questionnaire score [mean (SD)]^c^	2.6 (0.5)	2.7 (0.5)	2.6 (0.5)	2.5 (0.4)

	***n* (column%)**	***n* (row%)**	***n* (row%)**	***n* (row%)**

**Highest education**				
High school or less	70 (22)	54 (77)	7 (10)	9 (13)
Trade/vocational	37 (12)	22 (59)	1 (3)	14 (38)
Any university	205 (66)	129 (63)	36 (18)	40 (20)

**Main regular partner, partner HIV status**				
No	189 (60)	121 (64)	35 (19)	33 (17)
Yes, HIV-negative	79 (25)	52 (66)	7 (9)	20 (25)
Yes, HIV-positive or unknown status	46 (15)	34 (74)	2 (4)	10 (22)

**Receptive condomless sex with HIV+/unknown status regular or casual partner (previous 3 months)**				
Never	123 (39)	80 (65)	17 (14)	26 (21)
1 or 2 episodes	86 (27)	55 (64)	12 (14)	19 (22)
≥3 episodes	106 (34)	72 (68)	15 (14)	19 (18)

**Group sex (previous 3 months)**				
Never	98 (31)	68 (69)	16 (16)	14 (14)
1 or 2 episodes	99 (32)	59 (60)	18 (18)	22 (22)
≥3 episodes	116 (37)	80 (69)	9 (8)	27 (24)

**Crystal methamphetamine use (previous 3 months)**				
Never	173 (60)	108 (62)	29 (17)	36 (21)
Once per month	49 (17)	33 (67)	6 (12)	10 (20)
>Once per month	67 (23)	47 (70)	6 (9)	14 (21)

**Alcohol binge frequency (previous 30 days)**				
Never	84 (29)	53 (63)	16 (19)	15 (18)
Once per month	81 (28)	57 (70)	7 (9)	17 (21)
≥Twice per month	129 (44)	81 (63)	19 (15)	29 (22)

**Greatest HIV transmission risk**				
Main regular partner	58 (18)	42 (72)	8 (14)	8 (14)
Other regular partner	156 (50)	103 (66)	20 (13)	33 (21)
Casual partner	264 (84)	168 (64)	42 (16)	54 (20)
Group sex	167 (53)	105 (63)	25 (15)	37 (22)
Sex under the influence of drugs	145 (46)	98 (68)	17 (12)	30 (21)
Sex work	15 (5)	6 (40)	2 (13)	7 (47)

**First heard about PrEP**				
When invited into this study	20 (6)	14 (70)	2 (10)	4 (20)
<12 months before enrolment	178 (57)	120 (67)	27 (15)	31 (17)
>12 months before enrolment	117 (37)	73 (62)	15 (13)	29 (25)

**Site**				
Private Clinic	93 (30)	58 (62)	14 (15)	21 (23)
Public Clinic	222 (70)	149 (67)	30 (14)	43 (19)

*^a^Not all percentages add up to 100% due to rounding*.

*^b^Not every participant answered every question, so numbers may not always add up to 315*.

*^c^The transmission risk questionnaire score, HIV ARV questionnaire score, and self-efficacy questionnaire score were calculated as a mean score on a four-item Likert scale, with higher scores indicating greater agreement*.

No association was found between age and preferred PrEP dosing schedule (*p* = 0.304). There was, however, strong evidence of an association between highest level of education and preferred PrEP dosing schedule in the final multivariable model (*p* = 0.001). Participants with education beyond high school were more likely to indicate a preference for non-daily PrEP (*p* = 0.001). In particular, individuals with a trade/vocational qualification were more likely to prefer periodic to daily PrEP [aOR = 4.58, 95% CI: (1.68, 12.49)], compared with participants whose highest education was high school (Table 2).

**Table 2:** Predictors of preferences for event-driven or periodic (compared to daily) pre-exposure prophylaxis (PrEP) among gay and bisexual male participants (*n* = 315).

	Univariate—odds ratio (OR) [95% confidence interval (95% CI)]			Multivariate—adjusted OR (95% CI)		
	Reference category: daily PrEP			Reference category: daily PrEP		
	Event-driven	Periodic	*p*-Value	Event-driven	Periodic	*p*-Value
Age	1.02 (0.98, 1.05)	1.02 (0.99, 1.05)	0.304			

**Highest education**						
High school or less	Ref	Ref	0.003	Ref	Ref	0.001
Trade/vocational	0.35 (0.04, 3.02)	3.82 (1.44, 10.10)		0.38 (0.04, 3.34)	4.58 (1.68, 12.49)	
Any university	2.15 (0.90, 5.14)	1.86 (0.84, 4.10)		2.28 (0.94, 5.53)	2.04 (0.91, 4.56)	

**Main regular partner and partner HIV status**						
No	Ref	Ref	0.030	Ref	Ref	0.029
Yes, HIV−	0.47 (0.19, 1.12)	1.41 (0.74, 2.68)		0.45 (0.19, 1.09)	1.41 (0.72, 2.77)	
Yes, HIV+/unknown	0.20 (0.05, 0.89)	1.08 (0.48, 2.41)		0.20 (0.04, 0.87)	0.94 (0.41, 2.17)	

**High risk sex with regular or casual partner**
Never	Ref	Ref	0.962			
1 or 2 times in the last three months	1.03 (0.45, 2.32)	1.06 (0.54, 2.11)				
≥3 times in the last three months	0.98 (0.46, 2.10)	0.81 (0.41, 1.59)				

**Group sex**
Never	Ref	Ref	0.064			
1 or 2 times in the last three months	1.30 (0.61, 2.77)	1.81 (0.85, 3.85)				
≥3 times in the last three months	0.48 (0.20, 1.15)	1.64 (0.80, 3.37)				

**Crystal methamphetamine**						
Never	Ref	Ref	0.578			
Once per month	0.68 (0.26, 1.77)	0.91 (0.41, 2.03)				
>Once per month	0.48 (0.19, 1.22)	0.89 (0.44, 1.81)				

**Alcohol binge frequency**						
Never	Ref	Ref	0.359			
Once per month	0.41 (0.16, 1.07)	1.05 (0.48, 2.32)				
≥2 times per month	0.78 (0.37, 1.64)	1.27 (0.62, 2.58)				

**Beliefs and attitudes**
Greatest Transmission Risk: Partner	0.87 (0.38, 2.03)	0.56 (0.25, 1.27)	0.347			
Greatest Transmission Risk: Regular	0.84 (0.44, 1.62)	1.07 (0.61, 1.88)	0.818			
Greatest Transmission Risk: Casual	4.87 (1.13, 21.01)	1.25 (0.59, 2.68)	0.032			
Greatest Transmission Risk: Group sex	1.28 (0.66, 2.46)	1.33 (0.76, 2.34)	0.526			
Greatest Transmission Risk: Drug affected	0.70 (0.36, 1.36)	0.98 (0.56, 1.72)	0.565			
Greatest Transmission Risk: Sex work client	1.60 (0.31, 8.18)	4.11 (1.33, 12.73)	0.054			
Transmission Risk Questionnaire (mean)	1.51 (0.78, 2.90)	1.45 (0.83, 2.54)	0.244			
HIV anti-retroviral Questionnaire (mean)	0.83 (0.33, 2.11)	0.63 (0.29, 1.38)	0.510			
Self-efficacy Questionnaire (mean)	0.64 (0.31, 1.32)	0.45 (0.24, 0.85)	0.033	0.56 (0.26, 1.19)	0.39 (0.20, 0.76)	0.011

**First heard about PrEP**
When invited into this study	Ref	Ref	0.605			
In the last 12 months before this study	1.58 (0.34, 7.34)	0.90 (0.28, 2.94)				
More than 12 months before this study	1.44 (0.30, 7.00)	1.39 (0.42, 4.58)				

**Site**
Private Clinic	Ref	Ref	0.718			
Public Clinic	0.83 (0.41, 1.69)	0.80 (0.44, 1.46)				

Participants who reported having an HIV-positive main regular partner showed a strong preference for daily, compared to event-driven PrEP [aOR = 0.20, 95% CI: (0.04, 0.87)] in the final multivariate model (*p* = 0.029). There was no association between engaging in receptive CLAI with a regular or casual partner of HIV-positive or unknown status in the last 3 months and a preferred PrEP dosing schedule (*p* = 0.962). While neither crystal methamphetamine use (*p* = 0.578) nor alcohol binge frequency (*p* = 0.359) were significantly associated with a preferred PrEP schedule, there was a non-significant association between group sex and PrEP preference (*p* = 0.064).

In regards to the partner(s) participants believed presented the greatest HIV transmission risk, main regular (*p* = 0.347), other regular (*p* = 0.818), group sex (*p* = 0.526), and sex partners while under the influence of drugs or alcohol (*p* = 0.565) were not associated with a preferred PrEP dosing schedule. We found that participants who perceived casual partners as posing the greatest HIV transmission risk were significantly more likely to prefer event-driven PrEP (*p* = 0.032), while there was a trend for individuals who were concerned about contracting HIV from clients while engaging in sex work to prefer periodic PrEP (*p* = 0.054). However, these variables did not retain statistical significance in the final multivariate model.

Participants’ scores on the medication self-efficacy scale were significantly associated with a preferred PrEP dosing schedule (*p* = 0.011) in the final multivariate model. Participants who rated themselves better at taking medications were more likely to prefer daily over periodic PrEP [aOR = 0.39, 95% CI: (0.20, 0.76)]. However, neither the HIV transmission risk scale score (*p* = 0.244) nor the HIV ARV scale score (*p* = 0.510) were associated with a preferred PrEP dosing schedule. No association was found between a preferred PrEP dosing schedule and when participants first heard about PrEP (*p* = 0.605), or if they accessed PrEP through public or private health services (*p* = 0.718). The final multivariate model was tested for goodness-of-fit, and there was no evidence for lack of fit (*p* = 0.509).

## Discussion

One-third of GBM participants in the *PRELUDE* demonstration project in Sydney, Australia expressed a preference for non-daily PrEP regimens in their baseline online behavioural survey: 20 and 14% stated a preference for periodic and event-driven dosing, respectively. Higher levels of education were associated with a preference for periodic, compared to daily PrEP. Conversely, higher medication self-efficacy scores were associated with a preference for daily, as opposed to periodic, PrEP. Finally, participants with an HIV-positive main regular partner were more likely to prefer daily PrEP compared to event-driven PrEP.

Higher levels of education have previously been associated with greater acceptability and willingness to use PrEP (5, 26), as well as higher rates of PrEP use (13, 27), most notably in a similar cohort of early PrEP adopters in the United States (28). In the present study, where only daily PrEP was recommended to participants by clinicians, as per the Australian PrEP guidelines (23), participants with higher levels of education expressed a preference for periodic, compared to daily, PrEP. Given the established relationship between higher levels of education and better health literacy (29–31), it is understandable that individuals with more education may feel more confident in exploring non-daily PrEP regimens.

Interestingly, participants with greater belief in medication effectiveness and better relationships with their doctors and pharmacists were more likely to express a preference for daily PrEP. There was limited evidence for the efficacy of non-daily PrEP at the time of the baseline survey, and thus these participants may have simply trusted their clinicians’ recommendations for daily PrEP. Alternatively, this finding may be indicative of other events or practices occurring in participants’ lives, with one recent study noting that participants with more disruptive lives, such as those who engage in drug binges, believing they would be able to adhere to non-daily PrEP regimens better than committing to taking a daily pill (32). However, this was not seen in the HPTN 067 study, with participants in event-driven arm having significantly lower adherence and coverage of sex events compared to participants taking daily PrEP (15). Individuals should examine their own ability to take medications, and discuss any difficulties openly with their doctor, to ensure that they can maintain adequate levels of protection against HIV at the time of risk events—the key aim of PrEP use.

Intimate partner HIV status is also associated with participants’ preference for PrEP dosing schedule. Having an HIV-positive partner was associated with a preference for daily PrEP, perhaps due to the perceived ongoing nature of sexual risk in an HIV serodiscordant relationship. Although undetectable viral load was first proposed to prevent HIV transmission in 2008 (33), only recently has its effectiveness been confirmed for GBM in serodiscordant relationships (34). Given the scarcity of information about the efficacy of non-daily PrEP use at the time of conducting this survey, it is not surprising that participants in serodiscordant relationships expressed a preference for daily PrEP, as it was seen to afford the highest levels of protection against HIV.

We found no evidence that men with HIV-negative primary partners had a specific PrEP dosing preference, nor evidence of an association between PrEP preference and engaging in receptive CLAI with other regular or casual partners of HIV-positive or unknown status. While these partner types can pose a much higher risk of HIV transmission than an HIV-positive primary partner (35, 36), they may not be associated with the same prolonged anxiety or association with HIV. Thus, other HIV prevention strategies can be negotiated with other regular and casual partners, as compared with HIV-positive main regular partners.

Although crystal methamphetamine use was not found to be associated with a PrEP dosing preference in this study, methamphetamine use is known to be associated with higher risk of HIV acquisition (23, 37). A recent qualitative study of methamphetamine users found that frequency of drug use and participants’ ability to plan for sex were key determinants of PrEP dosing preference (32). Furthermore, methamphetamine use has been associated with poorer adherence to daily PrEP in injecting drug users (38), so non-daily regimens could be a more feasible form of HIV prevention and should be investigated further in this population.

Furthermore, no evidence was found in the final multivariate model between participants’ perceived greatest HIV transmission risk source and preferred dosing schedule. However, there was evidence in the univariate analysis that individuals who believed they were most at risk of HIV from sex with casual partners preferred event-driven PrEP regimens, while those who believed they were most at risk from sex work had a preference for periodic PrEP. These dosing preferences suit their risk patterns, as sex work is often seasonal or temporary (39), so individuals are not consistently at high risk of HIV. Similarly, individuals who are concerned about contracting HIV from their casual partners may only have CLAI on sporadic occasions and would prefer an event-driven schedule so that they could limit pill-taking to only around the time of sex.

With the exception of France (40), all countries to date that have approved oral PrEP or have guidelines pertaining to its use have done so only with respect to daily PrEP. However, the European AIDS Clinical Society (21) and World Health Organisation (41) guidelines, which are widely used, state that on-demand (or event-driven) PrEP is safe and effective in GBM. Thus, further evidence supporting the efficacy of non-daily PrEP regimens is still needed to inform regulatory bodies, clinicians, and PrEP users alike.

One key point to highlight is that a preferred PrEP dosing schedule does not necessarily translate to using PrEP in the manner described (42). This is particularly pertinent in a trial setting where adherence to daily PrEP was one of the measureable study outcomes. Investigating participants’ PrEP preferences at baseline then following how they take PrEP in a real-world setting, combined with collection of detailed attitudinal and behavioural data, would provide valuable insights into how people may change their PrEP dosing regimens dependent on other risk factors.

The timing of this study is also important to consider. These data were taken from the baseline survey, and most participants were enrolled in *PRELUDE* by the end of 2015. Although preliminary results from the IPERGAY study of event-driven PrEP were released earlier that year (43), dissemination of the findings into at-risk populations was somewhat limited. The lack of information and awareness in the community about non-daily PrEP regimens was compounded by clinicians recommending and prescribing all participants daily PrEP, all of which may have influenced participants’ decisions about which regimen was right for them. As more information becomes available about non-daily PrEP, it is increasingly pertinent that clinicians discuss dosing regimens with their patients to find the most suitable schedule, leading to improved adherence outcomes and reduced HIV infections as individually tailored HIV prevention strategies are adopted.

There are several caveats to this study. Most importantly, the relatively small number of participants expressing a preference for non-daily PrEP potentially limited the power to detect associations. Participants were also not asked about the cost of PrEP at this time, which has been previously shown to impact on PrEP willingness and use (44–46). Social desirability and recall biases may be present in behavioural survey data; however, participants completed the surveys in private after their visit and were ensured that clinical staff would not have access to their data, so any effects should have been limited. Despite these limitations, a number of factors were found to be strongly associated with preferences for particular PrEP dosing regimens. Furthermore, the sample consisted of predominately well-educated, health literate GBM at high risk of HIV, and thus our results may not be widely generalisable.

This study explored at-risk individuals’ preferences for non-daily PrEP and found that highest education level, intimate partner status, and participants’ self-reported medication self-efficacy were all strongly associated with a specific preference for PrEP dosing schedule. This research suggests that non-daily PrEP options may be chosen by substantial and growing sub-populations of people at high risk of HIV. Future research should investigate the translation of baseline preferences for PrEP into patterns of adherence, and how these may change with variations in behaviour over time.

## Prelude Study Team

**Andrew Carr**, St Vincent’s Hospital, NSW, Australia; **Andrew Grulich**, The Kirby Institute, UNSW Sydney, NSW, Australia; **Anna McNulty**, Sydney Sexual Health Centre, NSW Australia, and School of Public Health and Community Medicine, UNSW Sydney, NSW, Australia; **Brent Mackie**, AIDS Council of NSW (ACON), NSW, Australia; **Cathy Pell**, Taylor Square Private Clinic, NSW, Australia, and The Kirby Institute, UNSW Sydney, NSW, Australia; **Catriona Ooi**, Western Sydney Sexual Health Centre, NSW, Australia and Centre for Infectious Diseases and Microbiology, Westmead Clinical School, University of Sydney, NSW, Australia; **Ching (Yvonne) Cheung** (study clinical coordinator) - The Kirby Institute, UNSW Sydney, NSW, Australia; **Chris Gianacas** (clinical data manager), The Kirby Institute, UNSW Sydney, NSW, Australia; **David Templeton**, RPA Sexual Health, Sydney Local Health District and Central Clinical School, The University of Sydney, NSW, Australia, and The Kirby Institute, UNSW Sydney, NSW, Australia; **Dean Murphy**, The Kirby Institute, UNSW Sydney, NSW, Australia; **Edwina Wright**, Burnet Institute, VIC, Australia; **Garrett Prestage**, The Kirby Institute, UNSW Sydney, NSW, Australia; **Isobel (Mary) Poynten**, The Kirby Institute, UNSW Sydney, NSW, Australia; **John de Wit**, Centre for Social Research in Health, UNSW Sydney, NSW, Australia; **John Kaldor**, The Kirby Institute, UNSW Sydney, NSW, Australia; **John McAllister**, St Vincent’s Hospital, NSW, Australia; **Kenneth Mayer**, The Fenway Institute, MA, USA; **Mark Bloch**, Holdsworth House Medical Practice, NSW, Australia, and The Kirby Institute, UNSW Sydney, NSW, Australia; **Martin Holt**, Centre for Social Research in Health, UNSW Sydney, NSW, Australia; **Nathan Ryder**, Newcastle Community Health Centre, NSW, Australia; **Rebecca Guy**, The Kirby Institute, UNSW Sydney, NSW, Australia; **Rosalind Foster**, Clinic 16 Northern Sydney Sexual Health, NSW, Australia, and The Kirby Institute, UNSW Sydney, NSW, Australia; **Stefanie Vaccher** (behavioural data manager), The Kirby Institute, UNSW Sydney, NSW, Australia.

## Ethics Statement

All participants provided written informed consent before undertaking any study procedures, in accordance with the Declaration of Helsinki. The study was approved by St. Vincent’s Hospital Sydney Human Research Ethics Committee and registered under ClinicalTrials.gov (identifying number NCT02206555).

## Author Contributions

DT, MP, BH, CO, AG, and IZ were involved in the conception and design of the study. DT, CO, RF, and AM oversaw participant recruitment and data collection. SV and CG conducted the analysis. SV drafted the original manuscript. All authors contributed to the interpretation of data, revised the manuscript critically for important intellectual content, and approved the final version. All authors agreed to be accountable for all aspects of work.

## Conflict of Interest Statement

SV, CG, DT, MP, BH, CO, RF, and AM declare no competing interests. AG has received funding from BioCSL Ltd., Viiv and Gilead Sciences and has also received conference travel funding and honoraria for educational presentations from Merck. IZ has received funding and in-kind support (supply of the study medication) from Gilead Sciences for this study.

## Abbreviations

GBM, gay and bisexual men; PrEP, pre-exposure prophylaxis.
